# Supplementary material for: Potential Autoimmunity Resulting from Molecular Mimicry between SARS-CoV-2 Spike and Human Proteins
Source: Viruses. 2022 Jun 28;14(7):1415. doi: 10.3390/v14071415 (PMC9318917; doi:10.3390/v14071415)
Supplement: Supplementary file 1 [file viruses-14-01415-s001.zip › viruses-1729855-revision-supplementary_R2_no_track_changes.pdf]

Supplementary materials

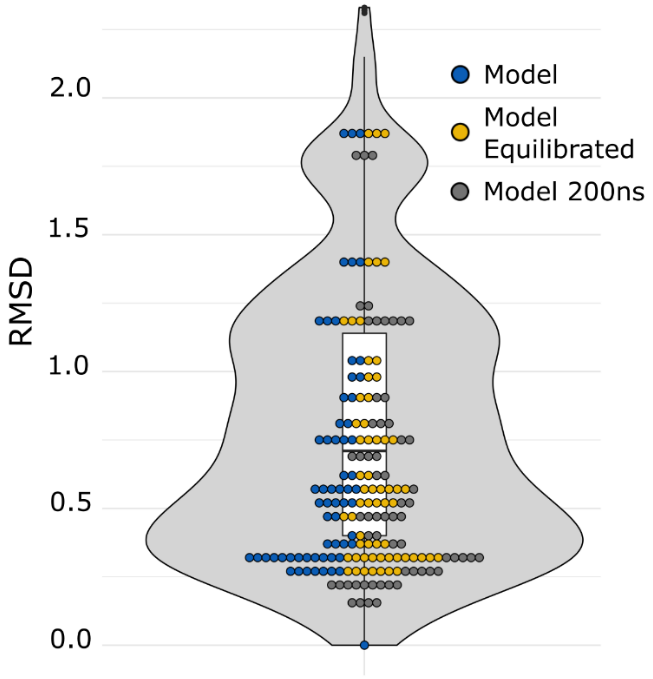

Figure S1. RMSD value distribution for solved and modeled Spike TQLPP regions. RMSD values resulting from an all-against-all comparison of the Spike TQLPP region of 63 structures, including the model in 3 states (shown as dots).

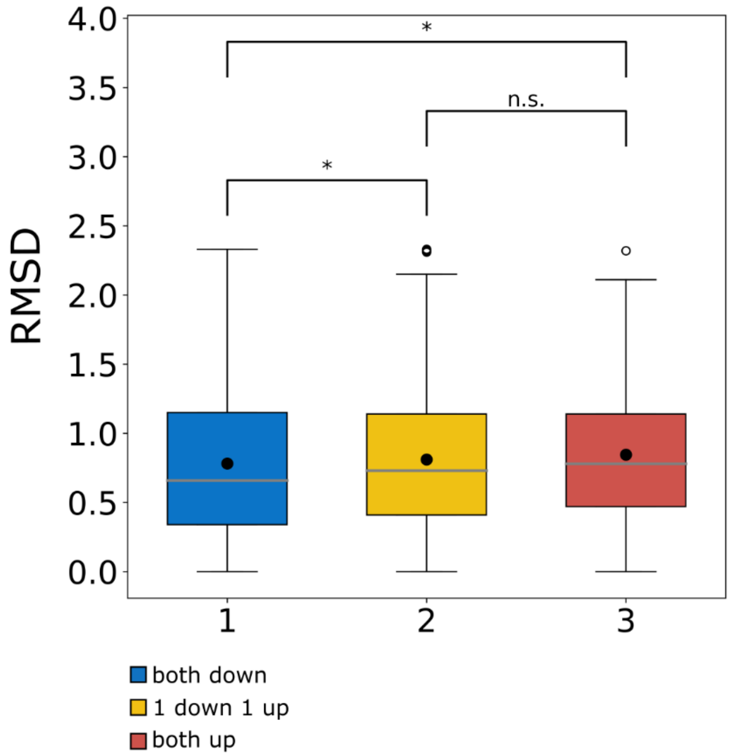

Figure S2. Comparison of RMSD values for TQLPP region from 20 Spike trimer structures based on RBD state. Box plots show distribution of RMSD values for Spike TQLPP where RBDs are: (1) both down (blue, N = 666, mean = 0.78 Å, median = 0.66 Å), (2) 1 down and 1 up (yellow, N = 962, mean = 0.81 Å, median = 0.73 Å), (3) both up (red, N = 325, mean = 0.85 Å, median = 0.78 Å). Statistical testing was performed using the Mann-Whitney U test. Brackets marked with an asterisk (*) denote statistically significant comparisons while those marked "n.s." denote non-significant comparisons. Groups 1 and 2 (p-value = 0.30) and 1 and 3 (p-value = 0.003) are significantly different but groups 2 and 3 (p-value = 0.055) are not. Box plots, bounded by the 1st and 3rd quartiles, show mean (black dot) and median values (horizontal solid gray line), vertical lines (whiskers) represent 1.5 × IQR, while outliers are marked as open circles.

a

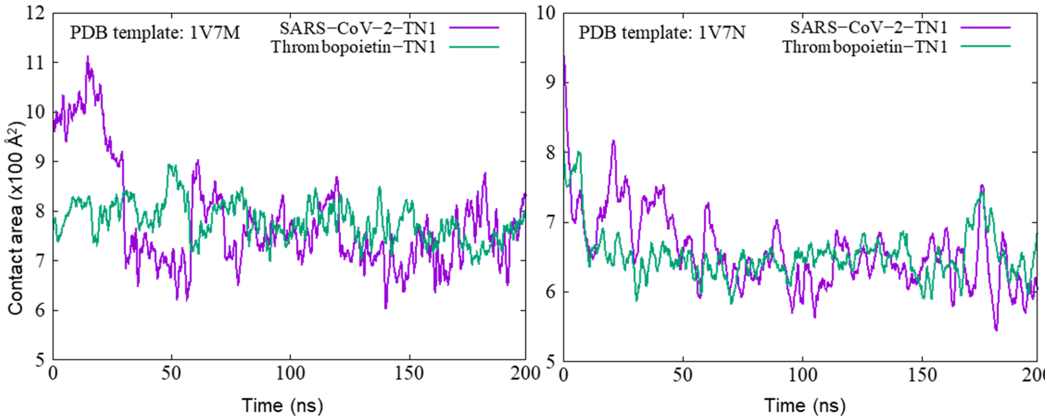

b

PDB template:

1V7M

hTPO \ TN1 →	D31	W33	W101	S102	S49	Y31	Y48	R90	R95
R98	**155**								
**Q111**	**55**	**76**		**85**					
**L112**			**81**						
Q61					**83**				
T58						**68**			
Q115							**53**		
**T110**								**44**	
G109									**30**

c

Run 1

Spike \ TN1 →	D31	W33	W101	K54	S30	S102
R237	**155**					
**Q23**	**48**	**78**				**10**
**L24**			**38**			
D111				**63**		
G72					**28**	
T73					**27**	

Run 2

Spike \ TN1 →	D31	W33	W101	S102	L53	Y48	N56
R21	**53**						
**Q23**	**15**	**78**		**72**			
**L24**			**67**				
Y28					**14**		
**P26**						**12**	
T20							**12**

d

PDB template:

1V7N

hTPO \ TN1 →	D31	W33	W101	S102	Y31	S49	L53
R98	**177**						
**Q111**	**29**	**84**		**62**			
**L112**			**73**				
E57					**43**		
Q61						**29**	
R115							**25**
T58					**15**		
**T110**				**13**			

e

Run 1

Spike \ TN1 →	D31	W33	W101	N56	K54	Y31
R21				**41**		
**Q23**	**65**	**78**				
**L24**			**74**			
D111					**10**	
T20				**22**		
S71						**9**

Run 2

Spike \ TN1 →	D31	W33	W101	K54	Y31
R21	**13**				
**Q23**	**22**	**81**			
**L24**			**77**		
D111				**30**	
V70					**18**

Figure S3. Molecular dynamics simulations overview. (**a**) Time evolution of the protein-antibody binding interface contact areas (100x Å^2) for Spike-TN1 (purple) and thrombopoietin-TN1 (green) in the molecular dynamics trajectories for PDB templates 1V7M (left) and 1V7N (right). Interaction matrices showing hydrogen bond contribution during the last 50 ns of 200 ns simulations between amino acid residue pairs ordered according to their hydrogen-bond

occupancies for the (**b, d**) hTPO-TN1 and (**c, e**) Spike-TN1 complexes for PDB template 1V7M and 1V7N, respectively. Residues belonging to TQLPP are colored in purple and positions for hTPO are based on the PDB template. TN1 Fab residues from heavy and light chains are shaded blue and yellow, respectively.

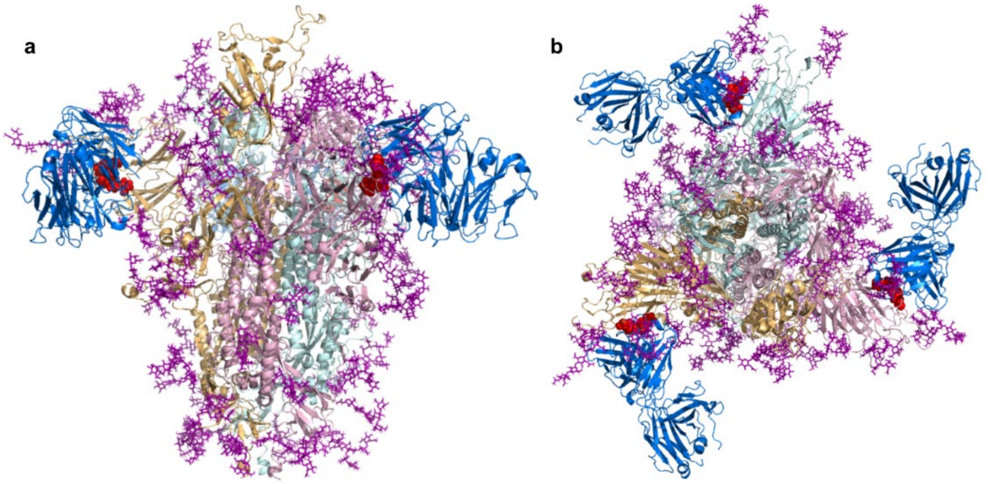

Figure S4. SARS-CoV-2 Spike bound to TN1 Fab antibody. SARS-CoV-2 Spike shown in the trimeric state (PDB id: 6VSB) bound to TN1 Fab antibody (blue, PDB id: 1V7M) as viewed from (**a**) the side and (**b**) the top. The TQLPP motifs are shown as red spheres and glycans are shown in purple. Structural visualization generated with PyMOL (Schrödinger, 2015).

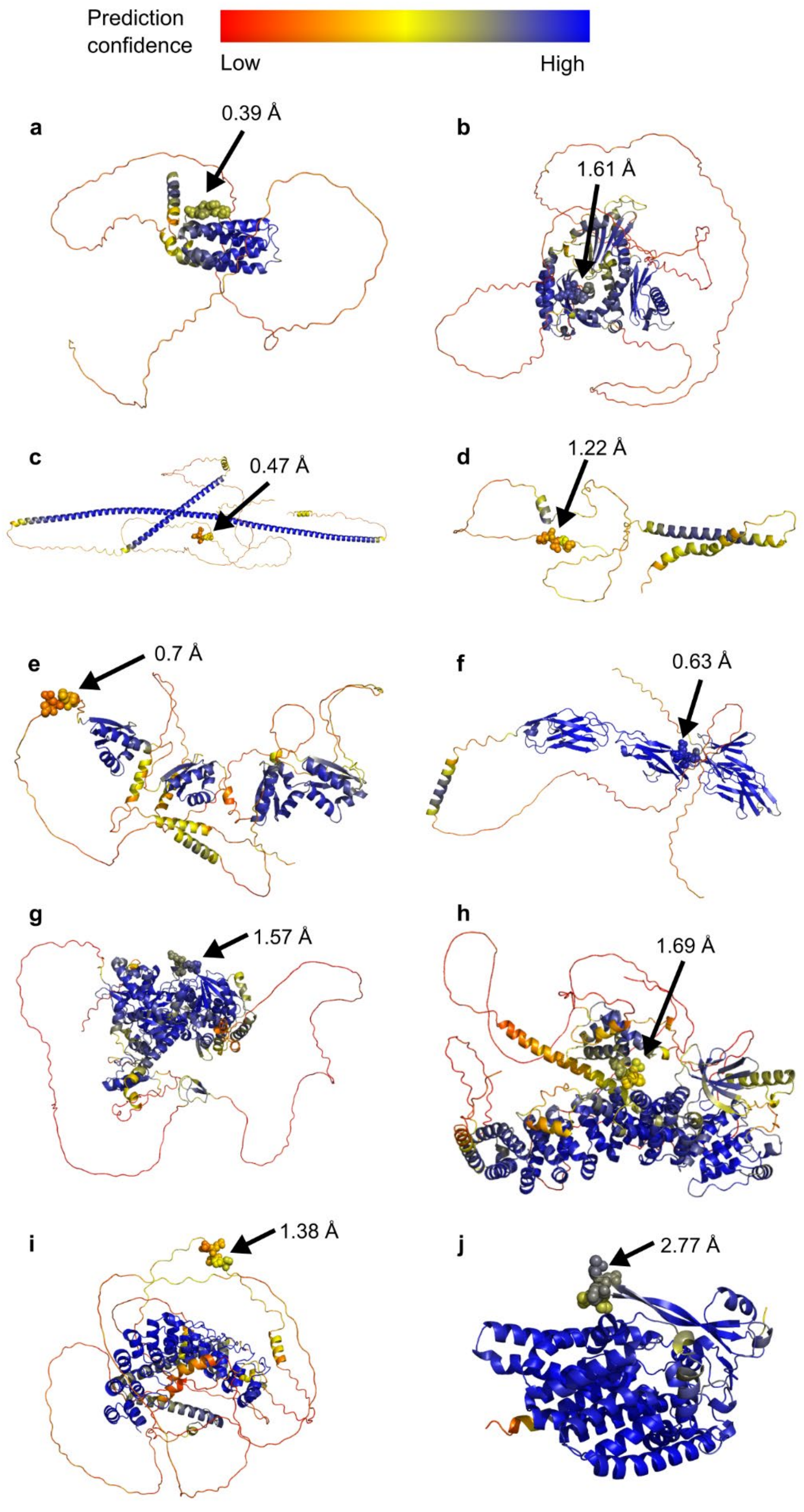
Prediction confidence
Low
High
a
0.39 Å
b
1.61 Å
c
0.47 Å
d
1.22 Å
e
0.7 Å
f
0.63 Å
g
1.57 Å
h
1.69 Å
i
1.38 Å
j
2.77 Å

Figure S5. TQLPP motif for 10 human proteins modeled by AlphaFold2. Protein structure models are colored by AlphaFold confidence estimate according to the color bar where red = 25 (low confidence) and blue = 100 (high confidence). TQLPP motif is shown as spheres. RMSD for human TQLPP in the 10 proteins compared to SARS-CoV-2 Spike (PDB id: 6XR8, chain A) is shown. The proteins are (**a**) thrombopoietin (Uniprot: P40225), (**b**) Hermansky-Pudlak syndrome 4 protein (Uniprot: Q9NQG7), (**c**) coiled-coil domain containing protein 85 (Uniprot: Q8N715), (**d**) transmembrane protein 52 precursor (Uniprot: Q8NDY8), (**e**) far upstream element-binding protein 1 (Uniprot: Q96AE4), (**f**) Fc receptor-like protein 4 (Uniprot: Q96PJ5), (**g**) DNA annealing helicase and endonuclease ZRANB3 (Uniprot: Q5FWF4), (**h**) serine/threonine-protein kinase NEK10 (Uniprot: Q6ZWH5), (**i**) espin (Uniprot: B1AK53), and (**j**) ALG12 (Mannosyltransferase ALG12 homolog, Uniprot: Q9BV10). Structural visualization generated with PyMOL (Schrödinger, 2015).

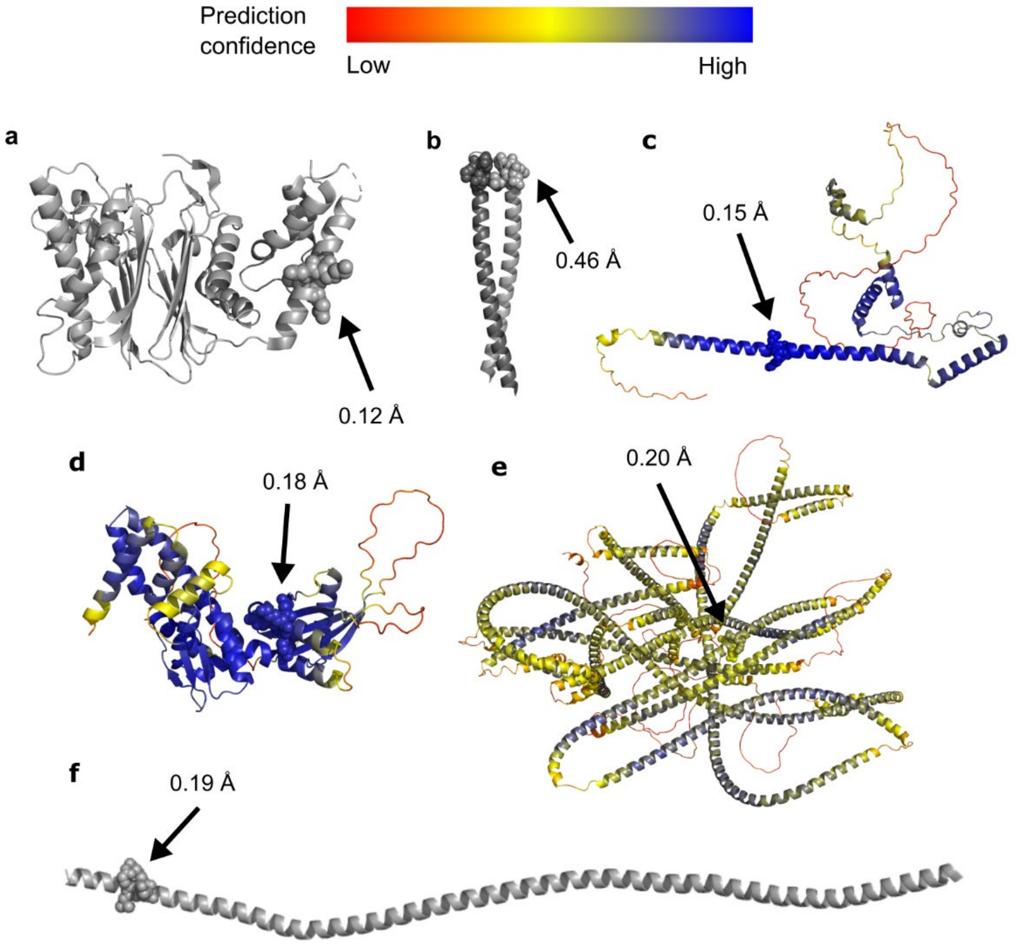

Figure S6. Structure of ELDKY motif for 5 human proteins. Protein structures from PDB are colored gray while AlphaFold2 3D models are colored by AlphaFold confidence estimate according to the color bar where red = 25 (low confidence) and blue = 100 (high confidence). ELDKY motif is shown as spheres. RMSD for human ELDKY in the 5 proteins compared to SARS-CoV-2 Spike (PDB id: 6XR8, chain A) is shown. The proteins are (**a**) protein phosphatase 1A (PDB id: 3FXJ), (**b**) leucine zipper domain of cGMP-dependent protein kinase 1 (PDB id: 3NMD), (**c**) protein FAM228B (Uniprot: P0C875), (**d**) protein Njmu-R1 (Uniprot: Q9HAS0), (**e**) thyroid receptor interacting protein 11 (Uniprot: Q15643), and (**f**) tropomyosin alpha-1 (PDB id: 6X5Z). Structural visualization generated with PyMOL (Schrödinger, 2015).

Table S1. RMSD values resulting from the alignment of the TQLPP region of 1V7M chain X and 1V7N chain X against the TQLPP region of 60 Spike structures. Sorted by RMSD.

1V7M X		1V7N X	
**Spike Structure**	**RMSD**	**Spike Structure**	**RMSD**
6ZGG A	0.36	7DCC E	0.21
6ZGG B	0.40	7DCC I	0.27
7BNN B	0.43	7DCC K	0.28
6ZGG C	0.44	7BNN B	0.42
7DCC E	0.44	7BNM C	0.44
7DCC I	0.48	7BNM B	0.44
7DCC K	0.48	7BNM A	0.44
7BNM A	0.52	7A25 C	0.46
7BNM B	0.52	6XR8 A	0.46
7BNM C	0.52	6ZGE C	0.47
7A25 C	0.59	6ZGE A	0.47
6ZGE A	0.60	6ZGE B	0.48
6ZGE B	0.60	6ZGG A	0.49
6ZGE C	0.60	7KMK B	0.49
7BNN A	0.60	7LRT B	0.49
6XR8 A	0.61	6ZGG C	0.49
7A25 A	0.63	7A25 A	0.50
7LRT B	0.66	6XR8 B	0.51
7LRT C	0.66	7LRT C	0.51
6XR8 B	0.68	6ZGG B	0.53
6XR8 C	0.71	6XR8 C	0.55
7A25 B	0.71	7A25 B	0.57
6ZP2 A	0.72	7LRT A	0.58
6ZP2 B	0.72	7N1U A	0.59
6ZP2 C	0.72	7KRQ A	0.61
7KMK B	0.72	7BNN A	0.61
7LRT A	0.73	7KRQ B	0.62
7KRQ A	0.76	7E8C A	0.64
7KRQ B	0.76	7BNN C	0.64
7N1U A	0.76	7KRQ C	0.66

7BNN C	0.78	7KMK C	0.68
7KRQ C	0.79	7E8C C	0.71
7E8C A	0.82	7E8C B	0.72
7LQV A	0.85	7N1U C	0.73
7KMK C	0.87	7JJI A	0.75
7N1U C	0.87	7JJI B	0.75
7E8C C	0.88	7JJI C	0.75
7LQV C	0.88	7N1U B	0.76
7E8C B	0.90	7KMK A	0.78
7N1U B	0.90	7LQV A	0.78
7LQV B	0.91	7LQV C	0.80
7JJI A	0.92	7LQV B	0.82
7JJI B	0.92	6ZP2 A	0.84
7JJI C	0.92	6ZP2 C	0.84
7CWL B	0.95	6ZP2 B	0.84
7CWS R	0.95	7MJG B	0.85
7KMK A	0.96	7MJG C	0.93
7MJG B	1.02	7MJG A	0.93
7MJG A	1.09	7CWL B	0.94
7MJG C	1.10	7CWS R	0.94
7CWL A	1.17	7CWS O	1.03
7CWS O	1.17	7CWL A	1.03
7C2L A	1.21	7C2L B	1.26
7C2L B	1.21	7C2L C	1.26
7C2L C	1.21	7C2L A	1.26
7N1Q B	1.62	7N1Q B	1.56
7CWL C	1.67	7CWS Q	1.57
7CWS Q	1.67	7CWL C	1.57
7N1Q A	1.68	7N1Q A	1.61
7N1Q C	1.71	7N1Q C	1.64

Table S2. RMSD values resulting from the alignment of the TQLPP region from 60 Spike structures and three modeled states, representing a conformational ensemble of TQLPP in Spike, sorted by RMSD.

Separate Excel sheet

Table S3. RMSD values for SARS-CoV-2 Spike wild-type TQLPP compared to corresponding region in known variants of concern.

VARIANT OF CONCERN	SPIKE PDB_CHAIN	RMSD (Å)	NOTES
**Alpha**	7N1U_A	0.21	
**Beta**	7N1Q_A	1.78	
**Gamma**	7SBS_A	1.17	P26S turns TQLPP to TQLPS
**Delta**	7SBK_A	0.69	
**Omicron BA.1**	7WE7_D	0.33	
**Omicron BA.2**	7UB0_A	N/A	Contains deletion of LPP
**Omicron BA.2.12.1**	Not available	N/A	
**Omicron BA.4**	Not available	N/A	
**Omicron BA.5**	Not available	N/A	

Table S4. PRODIGY binding affinities for antigen-antibody complexes

Complex	Frame	Binding Affinity (kcal/mol)	Intermolecular Contacts					
			Charged-Charged	Charged-Polar	Charged-Apolar	Polar-Polar	Polar-Apolar	Apolar-Apolar
**Spike-TN1 Fab (1V7M template)**	**1**	-11.5	3	18	11	20	35	19
	**2**	-8.6	0	5	2	7	16	20
	**3**	-9.2	2	8	8	6	15	16
	**4**	-8.8	3	6	3	7	15	14
	**5**	-9.7	3	7	8	10	20	22
	**Mean**	-9.56	2.2	8.8	6.4	10	20.2	18.2
	**Std Dev**	1.16	1.30	5.26	3.78	5.79	8.53	3.19
**Spike-TN1 Fab (1V7N template)**	**1**	-8.7	4	5	3	6	13	13
	**2**	-9.6	1	6	4	5	18	16
	**3**	-9.1	0	6	7	3	13	17
	**4**	-10.1	0	5	6	5	19	17
	**5**	-8.5	0	6	4	4	12	14
	**Mean**	-9.2	1	5.6	4.8	4.6	15	15.4
	**Std Dev**	0.66	1.73	0.55	1.64	1.14	3.24	1.82
**hTPO-TN1 Fab (1V7M)**	**1**	-9.2	1	11	9	10	20	16
	**2**	-10.3	1	9	10	7	21	20
	**3**	-9.2	1	9	9	7	17	16
	**4**	-9.5	2	10	8	9	20	15
	**5**	-9.3	3	8	6	9	20	18
	**Mean**	-9.5	1.6	9.4	8.4	8.4	19.6	17
	**Std Dev**	0.46	0.89	1.14	1.52	1.34	1.52	2.00
**hTPO-TN1 Fab (1V7N)**	**1**	-9.3	1	8	5	4	16	11
	**2**	-9.2	1	9	8	3	14	13
	**3**	-9.1	1	6	6	4	15	11
	**4**	-9.7	2	7	6	5	14	9
	**5**	-9.9	2	7	5	5	20	13
	**Mean**	-9.24	1.4	7.4	6	4.2	15.8	11.4
	**Std Dev**	0.43	0.55	1.14	1.22	0.84	2.49	1.67
**Spike-S2P6 Fab (7RNJ template)**	**1**	-9.5	2	5	10	0	10	13
	**2**	-9.2	2	6	8	0	10	16
	**3**	-9.6	1	5	11	0	11	16
	**4**	-9.9	1	6	11	0	11	17
	**5**	-9.4	2	5	10	0	11	16
	**Mean**	-9.52	1.6	5.4	10	0	10.6	15.6
	**Std Dev**	0.26	0.55	0.55	1.22	0.00	0.55	1.52

Table S5. Distribution of MaSIF binding confidence scores.

Separate Excel sheet

Table S6. Statistical comparison of MaSIF binding confidence scores for antibody complexes.

**MaSIF binding confidence scores**			
**Comparison**		**p-value**	**Significant** [1]
**Random**	Spike-Ab	4.83E-75	Yes
**Random**	hTPO-TN1	3.00E-08	Yes
**Random**	Spike-TN1	5.24E-26	Yes
**Spike-Ab**	hTPO-TN1	3.37E-02	No
**Spike-Ab**	Spike-TN1	7.68E-09	Yes
**hTPO-TN1**	Spike-TN1	1.92E-04	Yes

[1] Compared to Bonferroni corrected p-value (<8.33E-03) for alpha = 0.05

Table S7. RefSeq Select human isoforms that contain pentapeptides found in the 3D-mimics and AF-3D-mimics for SARS-CoV-2 Spike.

Separate Excel sheet

Table S8. MASIF binding confidence scores of other human proteins in complex with TN1.

UNIPROT ACCESSION[1]	PROTEIN NAME	MASIF BINDING CONFIDENCE SCORE	CONTACT PROTEIN[2]	CONTACT TN1
**Q6ZWH5**	NEK10	1.44195044	1047 GLN	102 SER
**Q9BV10**	ALG12	1.897805691	466 GLN	102 SER
**Q96PJ5**	FCRL4	2.539466143	215 GLN	102 SER

[1] Reference for AlphaFold2 prediction

[2] Corresponds to **Q** in T**Q**LPP
